# Nitrobenzylthioinosine mimics adenosine to attenuate the epileptiform discharge of hippocampal neurons from epileptic rats

**DOI:** 10.18632/oncotarget.16012

**Published:** 2017-03-08

**Authors:** Hao Huang, Jing Wang, Jun Zhang, Zhong Luo, Dongxu Li, Xiaowei Qiu, Yan Peng, Zhongxiang Xu, Ping Xu, Zucai Xu

**Affiliations:** ^1^ Department of Neurology, Affiliated Hospital of Zunyi Medical College, Zunyi, Guizhou, China; ^2^ Department of Prevention and Health Care, Affiliated Hospital of Zunyi Medical College, Zunyi, Guizhou, China

**Keywords:** epilepsy, nitrobenzylthioinosine, adenosine, adenosine A1 receptor, Neuroscience

## Abstract

Nitrobenzylthioinosine (NBTI), a specific inhibitor of type 1 equilibrative nucleoside transporter, could regulate the extracellular adenosine concentration and have protective roles in seizures. However, the protection mechanism of NBTI in seizures remains poorly understood. Here, the expression pattern and subcellular distribution of adenosine A1 receptor were detected by Western blot analysis and double-labeling immunofluorescence staining in Lithium Chloride-Pilocarpine induced epileptic rat model. At 24 h after pilocarpine induced rat seizures, hippocampal slices were prepared and the evoked excitatory postsynaptic currents (eEPSCs) amplitude of pyramidal neurons in hippocampus CA1 region was recorded using whole-cell patch clamp. *In vivo*, compared to control group, Western blotting analysis showed that the expression of adenosine A1 receptor protein was increased at 24 h and 72 h after seizure, didn't change at 0 min and 1 w, and decreased at 2 w. Double-label immunofluorescence revealed that adenosine A1 receptor was mainly expressed in the membrane and cytoplasm of neurons. *In Vitro*, adenosine decreased the eEPSCs amplitude of pyramidal neurons in hippocampus CA1 region, NBTI also had the same effect. Meantime, NBTI could further inhibit eEPSCs amplitude on the basis of lower concentration adenosine (50μM), and adenosine A1 receptor inhibitor DPCPX partially reversed this effect. Taken together, we confirmed that the expression of adenosine A1 receptor protein was increased in the early seizures and decreased in the late seizures. At the same time, NBTI mimics adenosine to attenuate the epileptiform discharge through adenosine A1 receptor, which might provide a novel therapeutic approach toward the control of epilepsy.

## INTRODUCTION

Epilepsy is a chronic brain disorder which characterized by recurrent unprovoked seizures and affects tens of millions of people in the world [1, 2]. High seizure frequency and various kinds of burdens are the main factors influencing the quality of life in epileptic patients [3, 4]. Increased seizure frequency is exactly associated with worse outcomes and the burdens of epilepsy. Of course, new antiepileptic drugs (AEDs) may reduce these adverse events and can improve the prognosis of patients with epilepsy [5]. However, the existing AEDs fail to prevent or cure the 20-30% of epileptic patients [6, 7].

Adenosine, an endogenous inhibitor of neuronal discharges [8], is released extensively during seizures, which could inhibit hippocampal glutamatergic synaptic transmission through interacting with pre- or postsynaptic adenosine A1 receptors [9–11]. At the same time, adenosine, as an endogenous anticonvulsant, could influence the stability of γ-aminobutyric acid type A receptor through the interaction with adenosine receptors [12]. Previous studies have shown that modulation and activation of adenosine A1 receptor could influence the frequency of spontaneous recurrent seizures and hippocampal excitability of chronic pilocarpine epileptic rats, and also have an anticonvulsant effect on seizures elicited from piriform cortex kindling by electrical stimulation [13, 14].

Type 1 equilibrative nucleoside transporter (ENT1) is one kind of most abundant and widely distributed in plasma membrane nucleoside transporter in mammalian cells and tissues [15, 16], and ENT1 is regarded as a critical transporter for the regulation of adenosine, and for the cellular uptake of chemotherapeutic nucleoside analogs [17, 18]. Previous study has illustrated that ectogenic adenosine A1 agonists that are transported by ENT1 could be applied to target central nervous system (CNS) disorders because of its widely distributed in the brain [19]. In addition, nitrobenzylthioinosine (NBTI) [20], a specific inhibitor of ENT1, could retard the adenosine disappearance from extracellular cleft through blocking adenosine uptake into cells, and has protective effects during seizures [21–23]. What is more, pretreated with NBTI could prolong the latency to reach the seizure status of animals [22, 23].

Based on the physiological roles of adenosine A1 receptor and NBTI in the CNS, we hypothesized that inhibition of glutamergic neurotransmission by adenosine via its A1 receptor may be mimicked by NBTI to attenuate the epileptiform discharge of hippocampal neurons from epileptic rats. This study was designed to determine the expression pattern and cellular distribution of adenosine A1 receptor in lithium chloride-pilocarpine induced epileptic rats using Western blot analysis and double-labeling immunofluorescence staining, and to evaluate the role of NBTI on the epileptiform discharge of hippocampus neurons from epileptic rats using the whole-cell patch clamp technique.

## RESULTS

### The expression of adenosine A1 receptor in the hippocampus of epileptic rats

**Figure 1:**
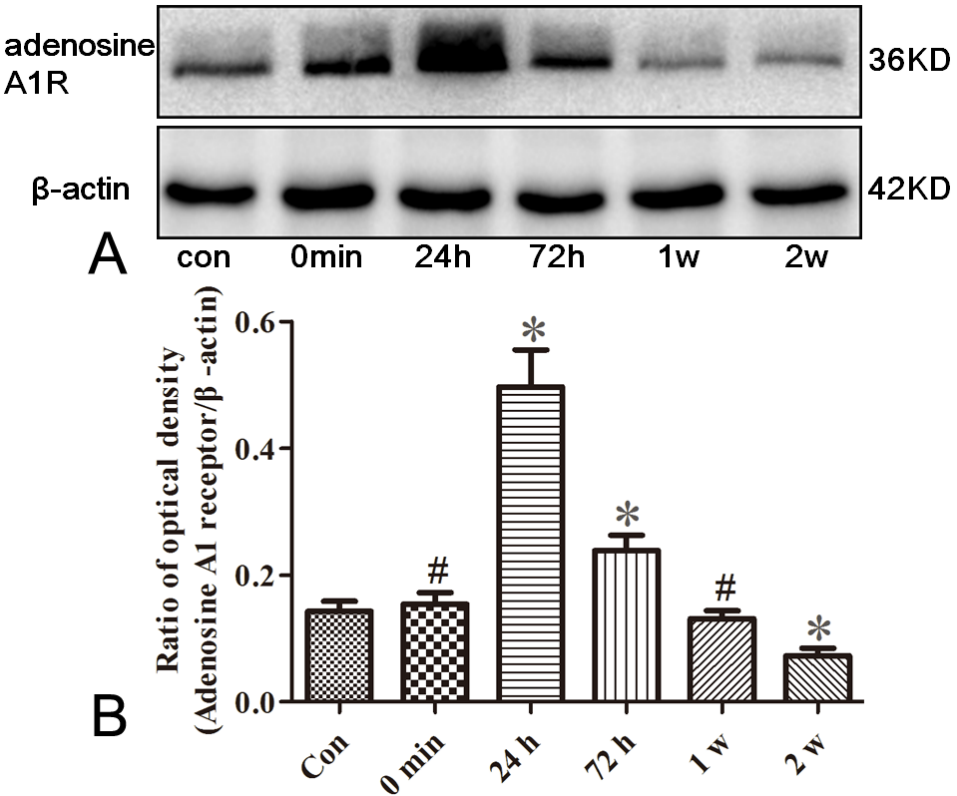
Western blotting analysis for adenosine A1 receptor in the hippocampus of rats The immunoreactive staining of adenosine A1 receptor in epileptic seizure rats are stronger than those in controls (Con) at 24 and 72 h after seizures, which are the same expression tendency as in controls at 0 min and 1 w time point. At 2 w after the onset of seizures, the adenosine A1 receptor expression is weaker than that in controls (*P < 0.01, ^#^ *P* > 0.05, *n* = 5).

To test whether adenosine A1 receptor was altered in rat seizure model, we measured dynamic changes of adenosine A1 receptor in the hippocampus of pilocarpine induced seizure rats. As shown in Figure 1A and 1B (*n* = 5), the adenosine A1 receptor expression, compared to control group (0.14±0.02), was peaked at 24 h (0.50±0.06) after seizure and remained significantly higher at 72 h (0.24±0.02, *P* < 0.05). However, the expression of adenosine A1 receptor at 0 min (0.15±0.02) and 1 w (0.13±0.01) had no significant difference compared with control group (*P* > 0.05). In addition, at 2 w (0.07±0.01) after seizures, the adenosine A1 receptor expression is weaker than that in controls (*P* < 0.05).

### Localization of adenosine A1 receptor in epileptic rats

**Figure 2:**
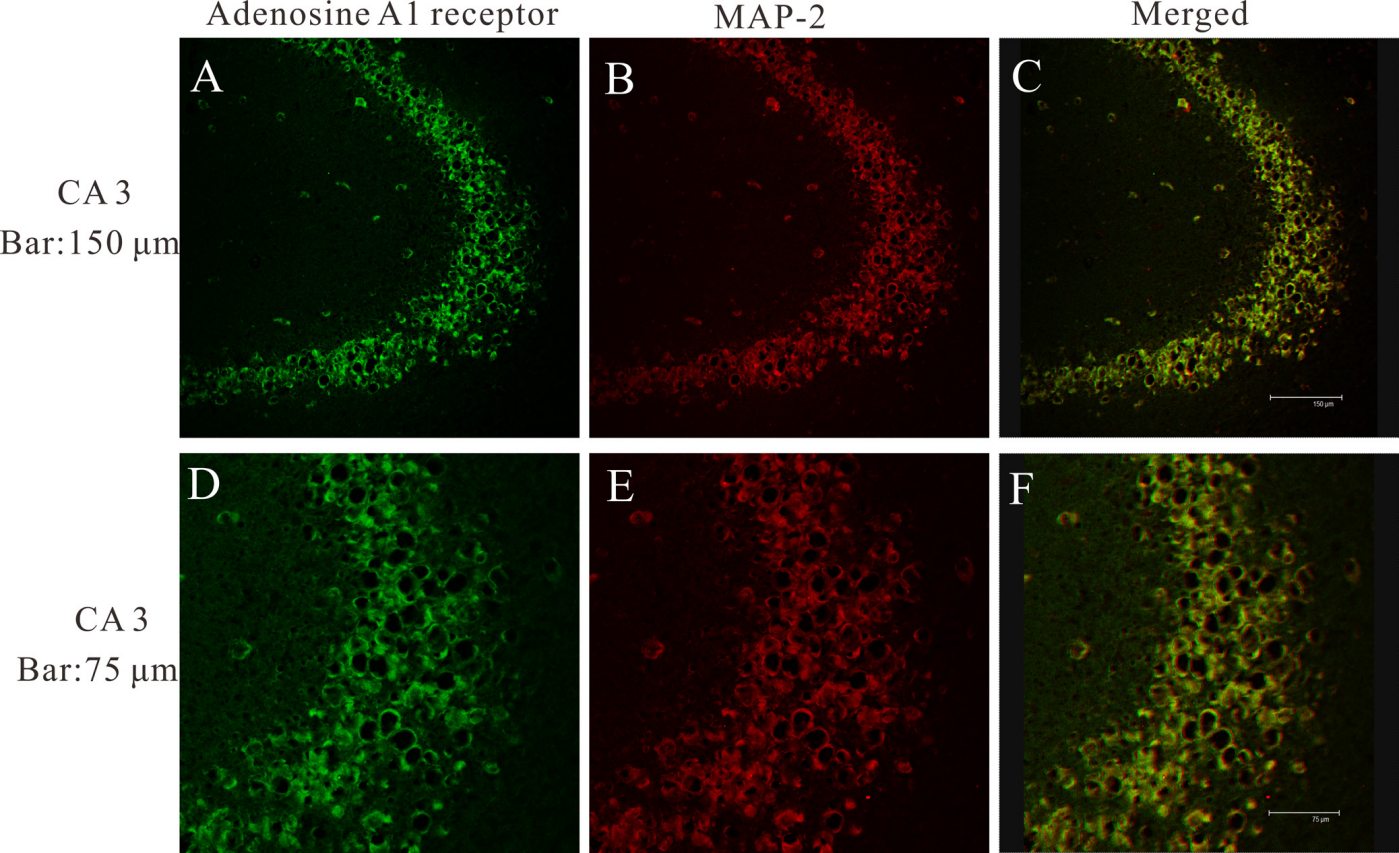
A laser scanning confocal image of adenosine A1 receptor in epileptic rat 24 h after seizures Adenosine A1 receptor (green) and MAP2 (red) are co-expressed within neurons in the CA3 area of hippocampus.

To determine the cellular distribution of adenosine A1 receptor, double-labeling immunofluorescence staining experiments were performed on the hippocampus of epileptic rats. At 24 h after seizures, we observed abundant immunoreactivity adenosine A1 receptor positive cells expressed in CA3 area of hippocampus (Figure 2A and 2D). Moreover, adenosine A1 receptor was colocalized with the neuron dendritic marker microtubule-associated protein 2 (MAP2) in the CA3 area of hippocampus (Figure 2C, 2F).

### NBTI mimics adenosine to inhibit eEPSCs mediated by adenosine A1 receptor

**Figure 3:**
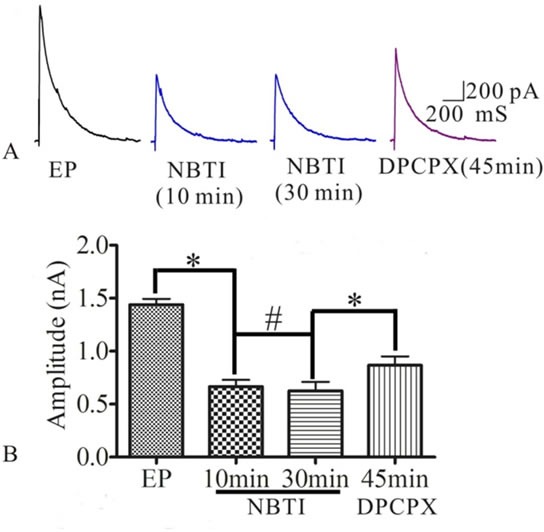
**A**. Respective eEPSCs in the presence or absence of NBTI (100 nM) or adenosine A1 receptor antagonist DPCPX (10 μM). 10, 30 and 45 min denote times after recording. **B**. Bar plot summary of eEPSC amplitude from A (* *P* < 0.01, ^#^ *P* > 0.05, *n* = 5).

**Figure 4:**
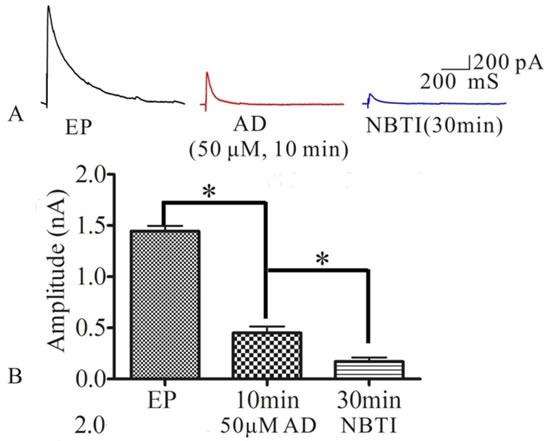
**A**. Respective eEPSCs in the presence or absence of AD (50μM) or NBTI (100 nM). 10 min and 30min denote times after recording. **B**. Bar plot summary of eEPSC amplitude from A (* *P* < 0.01, *n* = 5).

**Figure 5:**
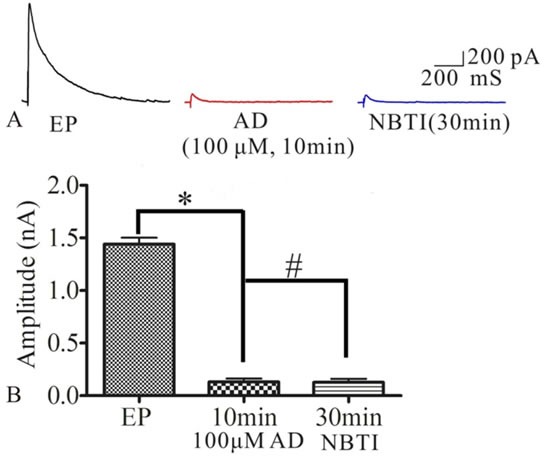
**A**. Respective eEPSCs in the presence or absence of AD (100μM) or NBTI (100 nM). 10 min and 30min denote times after recording. **B**. Bar plot summary of eEPSC amplitude from A (*P < 0.01, # *P* > 0.05, *n* = 5).

To test whether NBTI mimics adenosine effecting on eEPSCs mediated by adenosine A1 receptor, we investigated the role of adenosine and adenosine A1 receptor antagonist DPCPX in the process of NBTI regulating eEPSCs. At 24 h after pilocarpine induced rat seizures, preparation of hippocampal slices, selecting the pyramidal neurons of hippocampus CA1 region, the eEPSCs amplitudes were recorded by the whole-cell patch clamp, and they were stable during the whole recording process, which was consistent with previous study [24]. Compared to the eEPSCs amplitude of control epilepsy group (1.44±0.05), bath application of NBTI (100 nM) significantly reduced eEPSCs amplitude at 10 min (0.67±0.06) and 30 min (0.63±0.08), while application of A1 receptor inhibitor DPCPX (10 μM) partially reversed this effect (0.87±0.08, Figure 3A and 3B, n = 5). In addition, inhibition of eEPSCs amplitude by adenosine was mimicked by NBTI (100 nM), and the reduced eEPSCs amplitude by adenosine (50M, 0.45±0.06) were further inhibited by NBTI (100 nM, 0.17±0.04, Figure 4A and 4B, n = 5). However, when adenosine concentration was saturated (100μM, 0.15±0.02), no further inhibitory effect of NBTI on eEPSC amplitude was observed (0.15±0.02, Figure 5A and 5B, n = 5), so the inhibition of eEPSCs amplitude by adenosine was dose dependent. These results indicate that the effect of NBTI on eEPSCs was mediated by adenosine A1 receptor.

## DISCUSSION

In this study, we first measured adenosine A1 receptor expression in control and epileptic seizure rats. Then, we tested whether NBTI could mimic adenosine to attenuate the epileptiform discharge of epileptic hippocampus neurons. Interestingly, we observed that adenosine A1 receptor was mainly expressed in the membrane and cytoplasm of neurons, and the expression of adenosine A1 receptor protein was fluctuated, which increased at 24 h and 72 h after seizure, didn't change at 0 min and 1 w, and decreased at 2 w. At the same time, we observed that NBTI could mimic adenosine reducing eEPSCs amplitude through adenosine A1 receptor.

Adenosine, a well-known endogenous anticonvulsant modulate molecular, could regulate synaptic transmission and synaptic activity through interacting with adenosine A1 receptor [25, 26], while adenosine A1 receptor is closely related to neural network excitability [27, 28], and could reduce glutamatergic neurotransmitter release through inhibiting voltage-gated channels calcium ion influx [29], inhibit neuronal activity [25] and mediate seizure arrest [27, 30, 31]. So, based on the physiological roles of adenosine A1 receptor in the brain, adenosine A1 receptor may be associated with the pathogenesis of epilepsy.

In present study, we found that adenosine A1 receptor was mainly expressed in the membrane and cytoplasm of neurons, and the expression of adenosine A1 receptor protein was increased in the early seizures (24 h and 72 h after seizure), and decreased in the late seizures (2w after seizure). We speculate that increased adenosine A1 receptor protein in the early seizures may be related to the start of endogenous antiepileptic mechanism. Previous studies have shown that neural over activity in the early seizures induced the release of adenosine, which is an endogenous inhibitory neuromodulator and could mediates seizure arrest and limit the intensity or duration of seizures through interacting with adenosine A1 receptor [31–33]. While decreased adenosine A1 receptor protein expression in the late seizures may involve in epileptogenesis and recurrent seizures, which could consume endogenous antiepileptic substance. A growing number of studies have shown that adenosine A1 receptor has antiepileptic effects, adenosine A1 receptor agonists limits convulsions and seizures of experimental epileptic animals [34–37], while the antagonists of adenosine A1 receptor could worsen seizures [38]. Besides, spontaneous electrographic seizures were observed in the cerebral cortex of inactivated adenosine A1 receptor experimental epileptic mice [28], and in experimental traumatic brain injury (TBI) animal model, adenosine A1 receptor knockout promoted status epilepticus after TBI [39]. Moreover, modulation and activation of adenosine A1 receptors could influence the frequency of spontaneous recurrent seizures and hippocampal excitability of chronic pilocarpine-induced epileptic rats, and also have an anticonvulsant effect on seizures elicited from piriform cortex kindling by electrical stimulation [13, 14].

It is well-known that adenosine could inhibit the excitability of neurons through regulating synaptic transmission, synaptic activity and suppressing synchronous discharges [8, 25, 26, 40]. ENT1, one subtype of equilibrative nucleoside transporter, is regarded as a critical transporter for the regulation of adenosine and glutamatergic neurotransmission [18]. Under the condition of ischemia and hypoxia, ENT1 was over expressed and could reduce extracellular adenosine levels in mouse neurons [41], while ENT1 inhibitor (NBTI) could prolong onset latency for the development of seizures and status epilepticus in the epileptic animal models through increasing extracellular adenosine levels [21–23], and reduce evoked EPSC in lamina II neurons of rat spinal dorsal horn [24], suggesting that NBTI could reduce the excitability of neurons though adenosine signaling. Building on the earlier research, we have found that ENT1 played an important role in the epileptogenesis, and its specific inhibitor NBTI could attenuate seizure severity and prolong onset latency in pilocarpine-induced seizure rats [42]. In this study, we observed that adenosine decreased the eEPSCs amplitude of pyramidal neurons in hippocampus CA1 region at 24 h after pilocarpine-induced rat seizures, NBTI also had the same effect. Meantime, NBTI could further inhibit eEPSCs amplitude on the basis of lower concentration adenosine (50μM), and adenosine A1 receptor inhibitor DPCPX partially reversed this effect. So we thought that NBTI could mimic adenosine reducing eEPSCs amplitude through adenosine A1 receptor.

In conclusion, the abnormal expression of adenosine A1 receptor in the hippocampus of epileptic rats might be involved in the pathogenesis of epilepsy, NBTI mimics adenosine reducing epileptiform discharge through adenosine A1 receptor might provide a novel therapeutic approach toward the control of epilepsy.

## MATERIALS AND METHODS

### Epileptic model of rats

All experimental procedures were approved by the Commission of Zunyi Medical College for the Ethics of Experiments on Animals in accordance with China Animal Welfare Legislation standards. The adult male Sprague-Dawley (SD) rats weighing about 200 g were obtained from the Experimental Animal Center of Zunyi Medical College, China. The rats were maintained in a temperature controlled room (22-24°C) with a 12 h light and 12 h dark cycle as well as free access to food and water. The rat model of epilepsy was produced as before [42]. Briefly, male rats were intraperitoneal (i.p.) injected lithium chloride (127 mg/kg, Sigma-Aldrich Co., St. Louis, MO, USA) 20 h prior to the administration of pilocarpine (i.p. 50 mg/kg, Sigma-Aldrich). Furthermore, the rats were pretreated with atropine methyl nitrate (1 mg/kg) 30 min prior to pilocarpine administration. The rats received repeated i.p. injections of 10 mg/kg pilocarpine every 30 min until they developed epileptic seizures. The epileptic seizures were evaluated by Racine score [43]. Only convulsive seizures reached to the stage 4 or 5 were considered as being successfully kindled [44]. All rats were continuously monitored by a video recording system after injection of pilocarpine until they were sacrificed. The normal rats in control group were administration of an equal volume of saline instead of pilocarpine. The seizure rats were sacrifced at 0 min, 1, 3, 7 and 14 days after seizures, while the control group of rats were sacrifced at day 1, and the brain tissues were removed immediately for analysis (each group *n* = 5).

### Western blotting

Western blotting was performed as previous report to detect the expression of adenosine A1 receptor in normal and epileptic rats [45]. The rats’ hippocampal tissues were stored in liquid nitrogen for western blotting analysis. According to the manufacturers’ instructions, protein was extracted, and the protein concentration was measured by the Enhanced BCA Protein Assay Kit (Beyotime Institute of Biotechnology, Shanghai, China). A total of 50 μg protein was separated by 10% SDS-PAGE and then transferred to polyvinylidene fluoride membranes (Millipore Corp, Massachusetts, USA, 250 mA, 60 min). The primary antibodies were used as follows: rabbit anti-adenosine A1 receptor (1:500, Santa Cruz Biotechnology, CA) and rabbit anti-β-actin (1:1000, Beijing 4A Biotech Co., Ltd, Beijing, China). The membranes were washed 4 ×10 min with Tween-20/Tris-buffered saline and incubated with the appropriate diluted horseradish peroxidase-tagged secondary antibody (1:3,000, Santa Cruz Biotechnology, CA). The immunoreactive protein bands were visualized using an enhanced chemiluminescence substrate kit (Beyotime) before digital scanning (Bio-Rad Laboratories, California, USA). Blots intensities were calculated with the Quantity One software (Bio-Rad Laboratories, Hercules, CA, USA).

### Immunofluorescence

To detect the location of adenosine A1 receptor in hippocampus of epileptic rats, immunoreactivity of adenosine A1 receptor was observed by immunofluorescence. Brain hippocampus tissues was fixed in 4 % paraformaldehyde overnight at 4°C, then were respectively put in 20% and 30% graded sucrose solution for 48 h. Followed, 10-μm-thick frozen sections were cut on a freezing microtome and mounted on the polylysine-coated slides for immunofluorescence. The sections were dried at room temperature for 8 min and immersed in acetone for 15 min. After washed with PBS three times (5 min per time), the slices were heated in 0.01 M citric acid (pH 6.0) for 20 min at 92-98°C for antigen recovery. Then, the slices were permeabilized with 0.5 % Triton X-100 and incubated in normal goat serum (Zhongshan Golden Bridge, Inc., Beijing, China) for 30 min. Slices were incubated in rabbit anti adenosine A1 receptor (1:100; Santa) and mouse anti-MAP2 antibody (1:100; Boster, Wuhan, China) overnight at 4°C. After washed with PBS three times (5 min per time), the slices were incubated with anti-rabbit-FITC (green) and anti-mouse-TRITC (red) (1:100, Zhongshan Golden Bridge) for 60 min and mounted with 50 % glycerol and 50 % PBS in the dark. The fluorescence was detected by a laser scanning confocal microscopy (Leica, Heidelberg) on an Olympus IX70 inverted microscope (Olympus, Tokyo, Japan).

### Hippocampal brain slices preparation for Whole-cell patch clamp

According to previous report [42], the hippocampal brain slices for Whole-cell patch clamp were prepared as following. The SD rats were anesthetized by 3.5% chloral hydrate (1 ml/100 g, i.p.). The limbs were fixed, chest was opened and inferior vena cava was clamped. After cardiac perfusion by 0°C slices liquid (containing in mM: KCL, 2.5; NaH2PO4.2H2O, 1.25; MgCl_2_.H_2_O, 6; CaCl2, 1; NaHCO3, 26; sucrose, 220; glucose, 10), the whole brain was removed and placed in 0°C slice liquid for 3 min, then chopped into 350 μm thickness using an oscillating tissue slicer (Campden, NVSLM1), and were transferred to 37°C artificial cerebrospinal fluid (ACSF, in mM: NaCl, 124; KCl, 3; NaH_2_PO_4_.2H_2_O, 1.23; NaHCO_3_, 26; CaCl_2_, 2; MgCl_2_, 2; glucose, 10, buffered with 95%O_2_-5%CO_2_) for 1 h, then transferred to 23°C ACSF for 30 min.

### Whole-cell patch clamp recording

The following experiments were performed at 23°C. For recording the excitatory postsynaptic currents (EPSCs), a patch pipette (2-4 ΩM) was filled with intracellular solution (mM): 130 Cs-methanesulfonate, 10 HEPES, 10 CsCl, 4 NaCl, 1 MgCl_2_, 1 EGTA, 5 NMG, 5 MgATP, and 0.5 Na_2_GTP and 12 phosphocreatine, pH 7.2 (275-290 mOsm). Bicuculline (10μM) was added to block GABA mediated components in ACSF and record eEPSCs. In the eEPSCs recordings, a bipolar stainless steel electrode was placed in about 150 μm dorsolateral region of the recorded neurons, with a 40 μS, 60 μA and 0.1 Hz stimulus. Membrane potential was held at +40 mV. The signal was amplified using a Multiclamp 700B amplifier (Axon, USA) and digitized with a Digidata 1322A. Data were filtered at 10kHz and then recorded with pClamp 9.2 software (Molecular Devices, Sunnyvale, CA, USA).

### Statistical analysis

All data were expressed as mean ± standard error of mean (SEM), and the statistical analyses were performed with the SPSS 18.0, statistical comparisons were made by one-way ANOVA or Student's t test, *P* < 0.05 was considered significant.

## Abbreviations

AEDs: antiepileptic drugs
CNS: central nervous system
DPCPX: 3-dipropyl-8-cyclopentylxanthine
eEPSCs: evoked excitatory postsynaptic currents
ENT1: Type 1 equilibrative nucleoside transporter
i.p.: intraperitoneal
NBTI: nitrobenzylthioinosine
SD: Sprague-Dawley
TBI: traumatic brain injury
